# Glucose transporter 1 is important for the glycolytic metabolism of human endometrial stromal cells in hypoxic environment

**DOI:** 10.1016/j.heliyon.2020.e03985

**Published:** 2020-06-08

**Authors:** Takeharu Kido, Hiromi Murata, Akemi Nishigaki, Hiroaki Tsubokura, Shinnosuke Komiya, Naoko Kida, Maiko Kakita-Kobayashi, Yoji Hisamatsu, Tomoko Tsuzuki, Yoshiko Hashimoto, Hidetaka Okada

**Affiliations:** Department of Obstetrics and Gynecology, Kansai Medical University, Osaka, Japan

**Keywords:** Cell biology, Biological sciences, Molecular biology, Cell culture, Metabolomics, Oxidative stress, Reproductive system, Women's health, Endometrium, Hypoxia, Glycolysis, Glucose transporter, Human endometrial stromal cells

## Abstract

**Aim:**

The study aimed to elucidate the glycolytic metabolism of human endometrial stromal cells (hESCs) in hypoxic environment.

**Main methods:**

The hESCs were cultured in hypoxic environment, and their metabolic pathways were analyzed using metabolomics. We assessed glucose uptake using 2-deoxyglucose (2-DG) assay. The expression of glucose transporters (GLUTs) required for glucose uptake was determined using real-time quantitative polymerase chain reaction (qPCR) and western blotting. Furthermore, we knocked down GLUT1 and examined the uptake of 2-DG.

**Key findings:**

Under hypoxia, glucose-6-phosphate, fructose-6-phosphate, and fructose-1,6-diphosphate were significantly elevated in hESCs (P < 0.05). This finding indicated enhancement in glycolysis. The volume of glucose uptake increased significantly under hypoxia (P < 0.05). Hypoxia simultaneously induced the expression of GLUT1 and GLUT3 mRNA (P < 0.05) and attenuated the expression of GLUT8 (P < 0.05). Glucose uptake was significantly inhibited upon knockdown of GLUT1 (P < 0.0001).

**Significance:**

These results demonstrated a very important role of glucose transport under hypoxia. Also, hESCs utilize glycolysis to adapt to hypoxic conditions that could occur in menstrual and implantation period. These findings pave the way to study implantation failure and tumors originating from the endometrium.

## 1 Introduction

Menses occur normally at the end of each menstrual cycle, following the tissue breakdown and the loss of the majority of the endometrium (the functional layer). Previously, it was believed that menstruation completely exfoliates the functional layer and that uterus rejuvenation subsequently starts from the remaining basal layer, within a few days. However, it is now thought that some of tissue remains on the side of the basal layer, which contributes to the regeneration of functional layer [1]. During this time, the human endometrium suffers into hypoxic condition and causes spiral arteriole constriction [1]. Thus, the human endometrium has the capacity to adapt to hypoxia during menses [2].

Hypoxia-inducible factor 1α (HIF-1α) was identified in the human endometrium during menses using immunohistochemistry [3]. In vitro, hypoxia induces HIF-1α in human endometrial stromal cells (hESCs) [4]. HIF-1α is a transcription factor and the master regulator of the cellular response to hypoxia. Previous reports have shown that HIF-1α promotes cell survival under hypoxic condition by switching metabolism pathway from oxidative to glycolytic in different cells [5]. However, this metabolic pathway in the endometrium remains uncharacterized.

Metabolic activities and pathways produce various kinds of small molecules that are defined as metabolites and collectively constitute the metabolome. Metabolomics is a method developed to systematically analyze the metabolic fingerprint of a cell, tissue, organ, or organism [6]. Despite complete sequencing of numerous genomes is available, the functions of many genes are still unknown, and many catalyzing gene products are still unknown [7]. The metabolic analysis can be a powerful aid in demonstrating the activity of pathway because the metabolic pathway is well known in other cells [8].

Glucose is an essential metabolic substrate for most of the cells. Because glucose molecules are polar, they need special transport proteins to cross the biological membranes. Transport of glucose across cell membranes occurs by facilitated diffusion and is catalyzed by glucose transporters belonging to one of the facilitative glucose transporter superfamilies, which are membrane proteins found in most of the mammalian cells. Thus, in contrast to active transport proteins that require ATP to function and are restricted by low ATP/ADP ratio, facilitated diffusion transport proteins can function without consuming energy. Different glucose transporter isoforms play distinct roles in glucose metabolism, as defined by tissue expression patterns, substrate specificity, transport kinetics, and differential expression in diverse physiological situations [9]. Interestingly, HIF-1α increases glucose transporter 1 (GLUT1) expression [10]. Moreover, it was reported that hESCs express GLUT1, GLUT3, GLUT8, GLUT9, GLUT10, and GLUT12 [11,12].

Here, we hypothesized that hESCs survive the hypoxic environment by activating the metabolic pathways via GLUTs. This study aimed to elucidate the glycolytic metabolism and clarify the role of GLUTs in hESCs under hypoxic environment. We examined their metabolic pathways using metabolomics. We also investigated the expression of GLUT family members and measured glucose uptake in ESCs by silencing GLUT expression.

## 2 Methods

### 2.1 Tissue collection and culture of hESCs with hypoxic stimulus

All human tissues were obtained using a protocol for the protection of human subjects as approved by the ethical committee of Kansai Medical University and with informed consents from all the patients, in accordance with the Declaration of Helsinki. Human endometrial tissues were obtained from 17 patients, aged 32–47 years, who had undergone hysterectomies for the treatment of myoma uteri without hormonal therapy, and were in the proliferative phase, having regular menstrual cycles. Human ESCs were purified by the standard enzyme digestion method as described previously [13]. Cells were cultured in Dulbecco's modified Eagle's medium (DMEM)/F-12 medium supplemented with 10% fetal calf serum (FCS) (HyClone, Logan, UT, USA), 100 IU/ml penicillin and 100 mg/ml streptomycin (Invitrogen) at 37 °C under a humidified atmosphere containing 5% CO_2_ in air. The culture medium was replaced 30 min after plating to reduce epithelial cell contamination. The percentage of vimentin-positive cells in confluent hESCs was 99% as observed using immunohistochemical staining as described previously [14].

After passage 0–1, near confluency, the cells were trypsinized and re-plated in 6-well plates (1×10^6^ cells/well) for real-time quantitative polymerase chain reaction (qPCR) analyses and glucose uptake assay, or in 10 cm dishes (3.6×10^6^ cells/well under normoxia and 2.8×10^6^ cells/well under hypoxia) for metabolome analyses. To remove the effect of endogenous steroid hormones, cells were cultured until confluent and then the medium was replaced with phenol red-free DMEM/F12 supplemented with 10% dextran-coated charcoal stripped (DCS)-FCS. After 48 h, hESCs were washed and cultured in DCS-FCS containing echinomycin (a HIF-1α inhibitor; 100 nmol/L; ENZO Life Sciences, USA) [4] or dimethylsulfoxide as vehicle control under hypoxic (2% O_2_, 5% CO_2_, and 93% N_2_) and/or normoxic condition (5% CO_2_ and 95% air) for 24–48 h.

### 2.2 RNA extraction and real-time quantitative polymerase chain reaction analysis

Total RNA was extracted from cultured hESCs with RNeasy Minikit (Qiagen) according to the manufacturer's instructions. Real-time qPCR was carried out with the Thunderbird SYBR qPCR Mix (Toyobo) as described previously [15]. Real-time qPCR amplification of each sample was performed using respective gene primer pairs, together with primers for the elongation factor 1α (EF1α) gene, which is a valid internal control as reference gene for transcription profiling [16]. Primer sequences and accession numbers are shown in Table 1. Real-time qPCR of all the samples was set up with duplicate reactions, after which a melting curve analysis was carried out to monitor qPCR product purity. Relative expression levels were calculated for each sample after normalization against the housekeeping gene EF1α, using the ΔΔ threshold cycle (Ct) method for comparing relative fold expression differences [17].

**Table 1:** Primer sequences used for real-time PCR and amplicon sizes.

Target	Accession number	Forward primer 5′–3′	Reverse primer 5′–3′	Amplicon size (bp)
*GLUT1*	BC121804	TCCACGAGCATCTTCGAGA	ATACTGGAAGCACATGCCC	393
*GLUT3*	NM006931	ACTTTGACGGACAAGGGAAATG	ACCAGTGACAGCCAACAGG	180
*GLUT8*	NM014580	TCCGCTTTCTGCATCTTCAGT	CCCTCAAAATGGGCTGTGATT	95
*GLUT9*	NM020041	TCATCGCCTCTTTCTGCAGT	TGACGGTGCCTGCAATGAT	108
*GLUT10*	NM030777	GCCTTCTGCAACAGCTTCAAC	ACAAGCCGATGGTGCCAATG	82
*GLUT12*	NM145176	TGCTTGTTTATGTTGCTGCTTTTT	TGATCCCACCAGGAAAGATCTC	84
*VEGF*	X62568	CGAAACCATGAACTTTCTGC	CCTCAGTGGGCACACACTCC	302
*EF1α*	AF16869	TCTGGTTGGAATGGTGACAACATGC	AGAGCTTCACTCAAAGCTTCATGG	329

Note: GLUT = glucose transporter; VEGF = vascular endothelial growth factor; EF1α = elongation factor 1α.

### 2.3 Metabolome analysis

Culture medium was removed from the dish and cells were washed two times with 5% mannitol solution. The samples were then extracted using methanol containing an internal standard solution (Human Metabolome Technologies; HMT, Inc., Japan). Metabolome analysis was carried out by capillary electrophoresis electrospray ionization time-of-flight mass spectrometry and capillary electrophoresis-triple quadrupole mass spectrometry at Human Metabolome Technologies, Inc. (HMT, Inc., Japan) (n = 3 in each group) [18, 19].

### 2.4 GLUT1 silencing with small interfering RNA

To silence the GLUT1 expression, two small interfering RNAs (siRNAs) directed against distinct areas of the gene sequence were designed (catalog no. HSS109811 [GLUT1-A] and HSS109812 [GLUT1-B], Human Stealth Select RNAi; Invitrogen). Non-silencing RNA (catalog no. HSS12935-112) was used as negative control. Cells were cultured until 30% confluency, as determined by the experimental condition optimization, and then were transfected with each siRNA (10 nmol/L) using Lipofectamine RNAiMAX transfection reagent (Invitrogen) according to the manufacturer's instructions. The hESCs were transfected for 2 days and were then changed to DCS-FCS, cultured under hypoxia or normoxia. After 24 h, the medium was removed and RNA was extracted from cells. The silencing of the GLUT1 gene was confirmed by real-time qPCR and western blotting analysis. Each experiment was repeated at least 3 times with different cell preparations.

### 2.5 Western blotting analysis

Cultured cells were homogenized in lysis buffer containing Mammalian Protein Extraction Reagent (Thermo Fisher Scientific Inc.) and protease inhibitor cocktail. Samples were divided and loaded on precast polyacrylamide gel (Bio-Rad, Laboratories, Inc.) for GLUT1, GLUT3 and β-actin and then transferred to Immuno-Blot polyvinylidene difluoride membrane (Bio-Rad). Non-specific-binding sites were blocked using 5% skim milk powder in Tris-buffered saline for 1 h at room temperature. Blots were then incubated overnight at 4 °C with rabbit polyclonal GLUT1 antibody (1:1000; abcam), rabbit polyclonal GLUT3 antibody (1:400; abcam) or mouse monoclonal β-actin antibody (1:5000; Sigma-Aldrich) as primary antibody; and later with anti-mouse immunoglobulin (Ig) G peroxidase-labeled secondary antibody (1:10000; GE Healthcare Life Science) or anti-rabbit IgG peroxidase-labeled secondary antibody (1:10000; Invitrogen), respectively. Immune complexes were visualized by enhanced chemiluminescence plus western blotting detection reagents (GE Healthcare Life Science) and visualized using FUSION Solo S (Vilber Lourmat). The protein levels were evaluated using ImageJ software. ImageJ is a public domain image-processing program developed by the National Institutes of Health and is freely available at http://rsbweb.nih.gov/ij. Fold increase was computed by dividing the relative expression of GLUT1 and GLUT3 by the relative expression of β-actin.

### 2.6 Glucose uptake assay

The volume of glucose uptake in hESCs was measured using 2-deoxyglucose (2-DG) uptake assay kit (Cosmobio) as previously described [20]. Cells were placed in serum-free medium for 6 h. Then, the cells were rinsed three times with Krebs Ringer Phosphate Hepes (KRPH) buffer (1.2 mM KH_2_PO_4_; 1.2 mM MgSO_4_; 1.3 mM CaCl_2_; 118 mM NaCl; 5 mM KCl; 30 mM Hepes, pH 7.5). ESCs were treated for 20 min at 37 °C with KRPH buffer supplemented with 2% BSA and 1 mM 2-DG in the absence and presence of 40 μM cytochalasin B to correct for non-specific uptake. Glucose uptake was stopped by washing the cells three times in ice-cold phosphate-buffered saline (PBS) without calcium and magnesium. The samples solubilized using ultrasonic homogenizer (MISONIX ASTRASON XL2020) in 10 mM Tris-HCl (pH 8.0) were heated for 15 min at 80 °C and centrifuged at 15000× g for 20 min at 4 °C. Next, the 2-DG uptake volume of the upper layer was measured according to the manufacturer's instructions. When 2-DG is taken up by the cells, it is phosphorylated to 2-DG 6-phosphate (2DG6P). Nicotinamide adenine dinucleotide phosphate (NADPH) is produced in the proportion to 2DG6P. This system detects NADPH by enzyme cycling method.

### 2.7 Cell counting kit-8

Proliferation assays were carried out using a cell counting kit-8 containing WST-8 (Dojin Laboratories, Japan), according to the manufacturer's instructions as described previously [21]. After hESCs were transfected with siRNA to silence GLUT1 expression for 2 days, and cells were cultured under hypoxia or normoxia for 24 h. WST-8 (10μL) was added and then readings were taken at 450 nm. Each experiment was done in triplicate.

### 2.8 Cell death detection ELISA

Apoptosis of hESCs was quantified by direct determination of nucleosomal DNA fragmentation by a Cell Death Detection ELISA according to the manufactures’ protocol (Roche Diagnostics GmbH, Mannheim, Germany). Briefly, cell lysates were obtained from hESCs treated with siRNA for 2 days to silence GLUT1 expression, and cultured under hypoxia or normoxia for 24 h. The substrate solution was added to each well and the absorbance was read at 405 and 492 nm as a reference.

### 2.9 Statistical analysis

Values are expressed as mean ± SEM. Differences in the measured parameters across the different groups were assessed by multiple comparison correction performed with the Tukey-Kramer procedure (JMP 12.2.0; SAS Institute, Cary, NC). Metabolome was analyzed with the Welch's t-test comparing the means of metabolite concentrations. Other two-group analyses were performed using Student t-test. All p values < 0.05 were considered statistically significant.

## 3 Results

### 3.1 Hypoxia induces metabolic changes in hESCs

**Figure 1:**
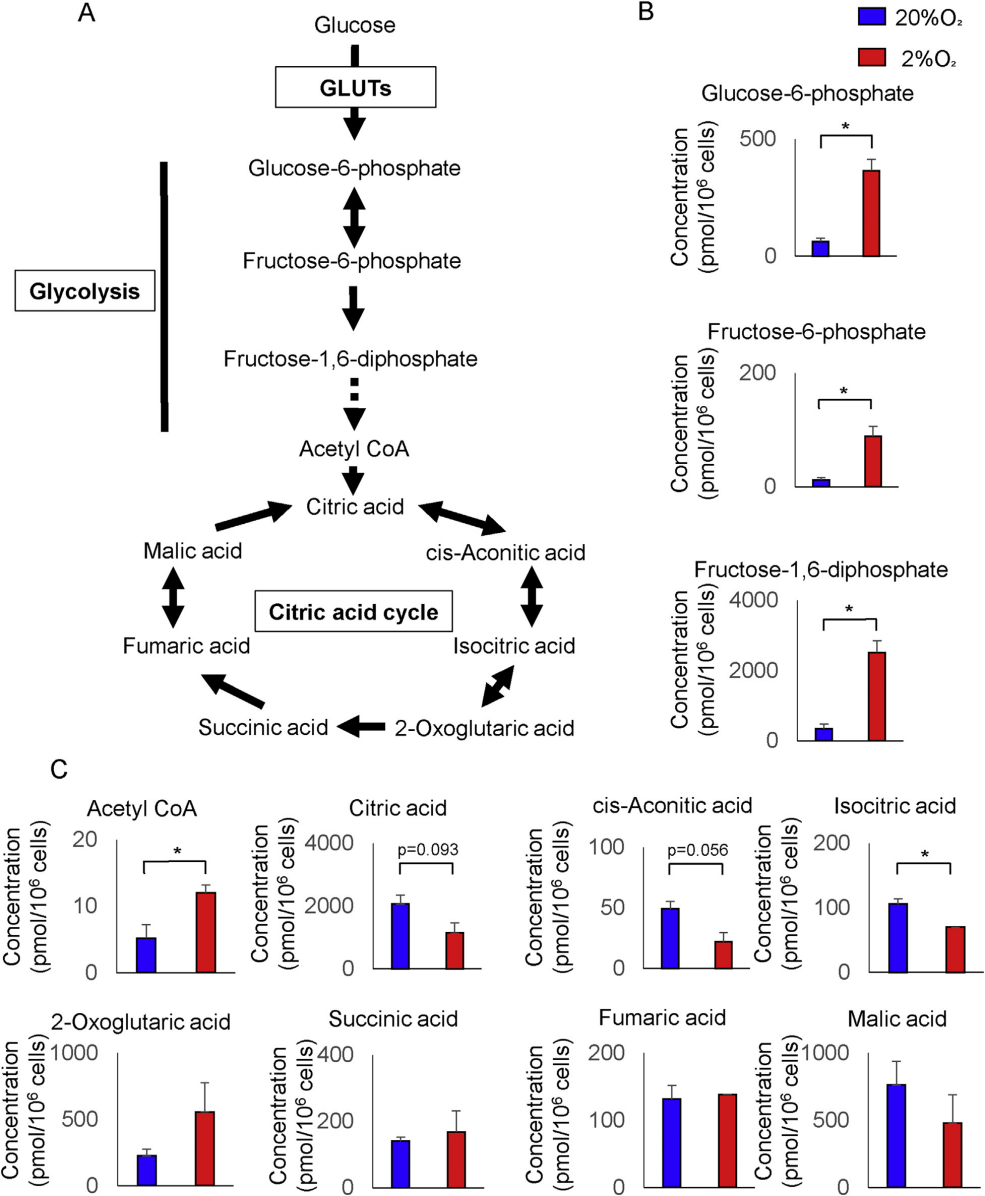
Metabolic pathway in human endometrial stromal cells (hESCs). (A) A typical schematic of metabolic pathway. Glucose enters hESC through glucose transporters (GLUTs) and is converted to glucose-6-phosphate, fructose-6-phosphate, and fructose-1,6-diphosphate by glycolytic enzymes. Citric acid cycle intermediates are acetyl CoA, citric acid, cis-aconitic acid, isocitric acid, 2-oxoglutaric acid, succinic acid, fumaric acid, and malic acid. The hESCs were cultured under hypoxia (2% O_2_) or normoxia (20% O_2_) for 24 h before analysis. (B) Shown is the measured volume of metabolites involved in the glycolysis. (C) Shown is the measured volume of metabolites involved in the citric acid cycle. Results are combined data of three experiments with different cell preparations and each value represents mean ± SD; ∗P < 0.05.

**Figure 2:**
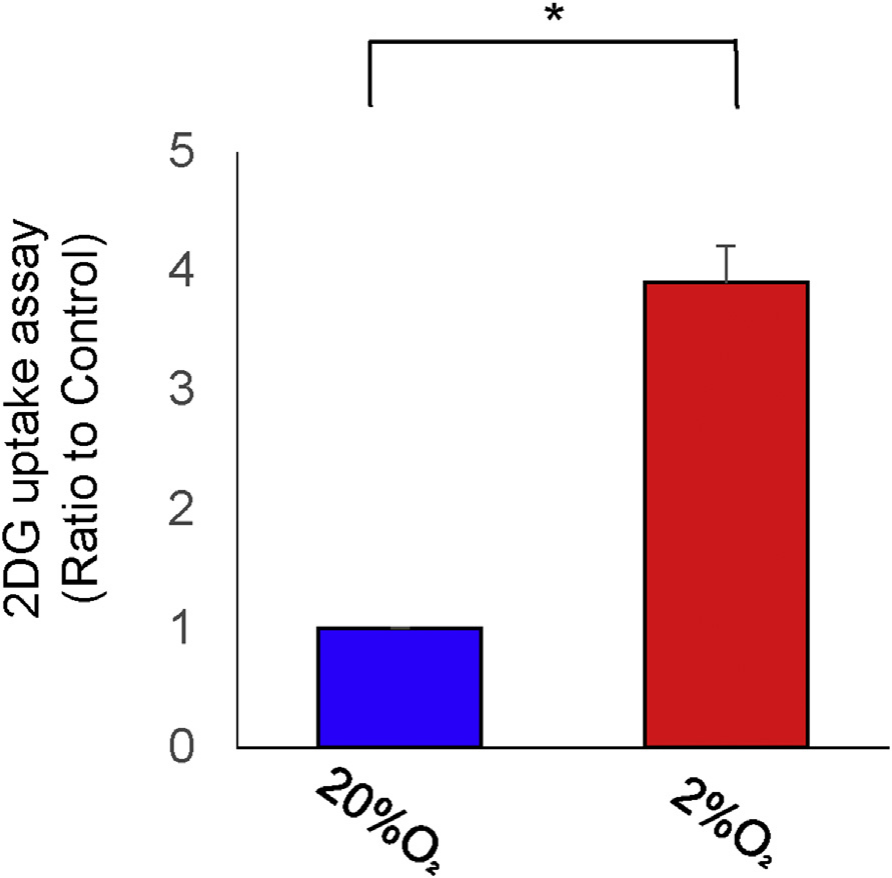
The effect of hypoxia treatment on glucose uptake in human endometrial stromal cells (hESCs). Cells were cultured under hypoxia (2% O_2_) or normoxia (20% O_2_) and then incubated with the 2-deoxyglucose (2-DG) for 20 min. Results are combined data of three experiments with different cell preparations and each value represents mean ± SEM; ∗P < 0.05.

To characterize the effects of hypoxia on energy metabolic pathways, a total of 18 metabolites were analyzed (Table 2). Several metabolic intermediates in glycolysis (glucose-6-phosphate, fructose-6-phosphate, fructose-1,6-diphosphate; see Figure 1A) showed a remarkable increase under hypoxia compared to normoxia (Figure 1B; P < 0.05). Moreover, hypoxia significantly reduced the isocitric acid and tended to suppress cis-aconitic and citric acid, which are important metabolites in the tricarboxylic acid cycle (Figure 1C). The results showed a significant increase in acetyl CoA (Figure 1C; P < 0.05). These findings suggest that hypoxia simultaneously stimulates glycolysis and suppresses tricarboxylic acid cycle. To test glycolytic substrates, we further measured the uptake of extracellular glucose using a 2-DG uptake assay. As shown, hESCs under hypoxia showed a significantly higher glucose uptake than under normoxia (Figure 2; P < 0.05).

**Table 2:** The concentration of 18 metabolites.

Compound name	Concentration (pmol/10^6^ cells)				p-value
	Control		Hypoxia		
	Mean	SD	Mean	SD	
Glucose-6-phosphate	63	24	365	84	0.019
Fructose-6-phosphate	12	6.6	89	30	0.043
Fructose-1,6-diphosphate	351	227	2522	576	0.013
Glyceraldehyde-3-phosphate	65	48	375	256	0.167
Dihydroxyacetone phosphate	195	136	875	516	0.142
3-Phosphoglyceric acid	91	9.1	88	26	0.860
2-Phosphoglyceric acid	9.1	1.3	9.4	2.5	0.850
Phosphoenolpyruvic acid	23	2.4	19	7.0	0.431
Pyruvic acid	160	37	279	156	0.316
Lactic acid	18694	2008	50271	17180	0.084
Acetyl CoA	5.2	2.0	12	1.2	0.014
Citric acid	2063	479	1144	538	0.093
cis-Aconitic acid	49	11	22	13	0.056
Isocitric acid	106	14	70	0.6	0.048
2-Oxoglutaric acid	226	81	555	375	0.266
Succinic acid	141	20	168	109	0.712
Fumaric acid	131	34	138	Not Available	Not Available
Malic acid	761	296	476	358	0.453

**Figure 3:**
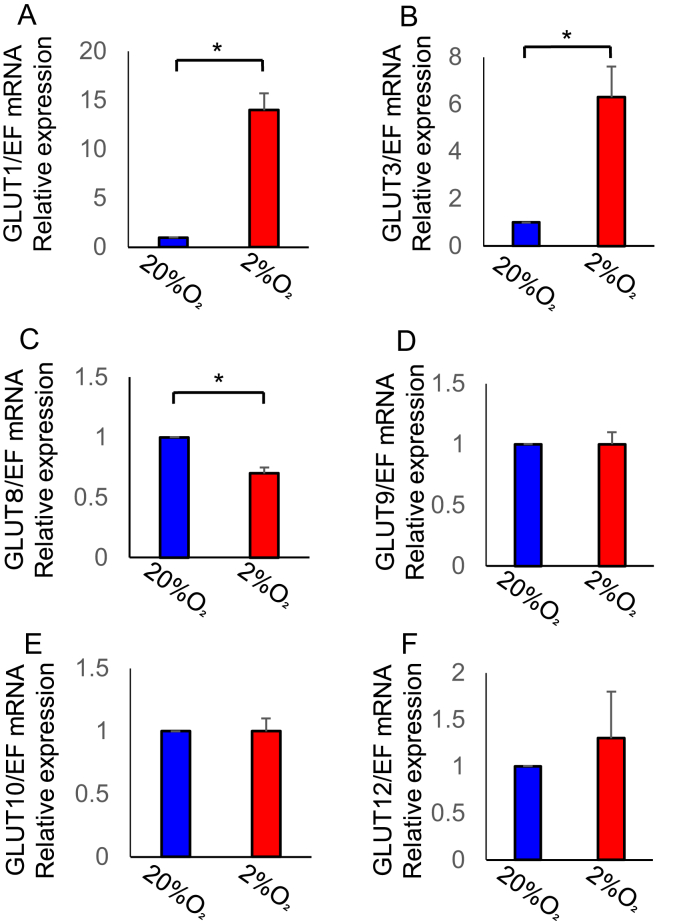
Effects of hypoxia on glucose transporters (GLUTs) expression in human endometrial stromal cells (hESCs). Cells were cultured under hypoxia (2% O_2_) or normoxia (20% O_2_) for 24 h. The mRNA levels of GLUT1 (A), GLUT3 (B), GLUT8 (C), GLUT9 (D), GLUT10 (E), and GLUT12 (F) were analyzed by real-time qPCR and normalized to elongation factor1α (EF1α) mRNA expression. Results are combined data of four experiments with different cell preparations and each value represents mean ± SEM; ∗P < 0.05.

Next, we investigated the expression of GLUTs (GLUT1, GLUT3, GLUT8, GLUT9, GLUT10, and GLUT12 existing in hESCs), which transport the glucose intracellularly. Real-time qPCR analysis confirmed that hypoxia increased the expression of GLUT1 and GLUT3 while decreasing the expression of GLUT8 (Figure 3; P < 0.05). GLUT1 is involved in glucose uptake under hypoxia, because GLUT1 was induced by hypoxia and was the most abundant in hESCs compared to other GLUTs [12].

### 3.2 Effects of echinomycin on GLUT1 expression mediated through HIF-1α

**Figure 4:**
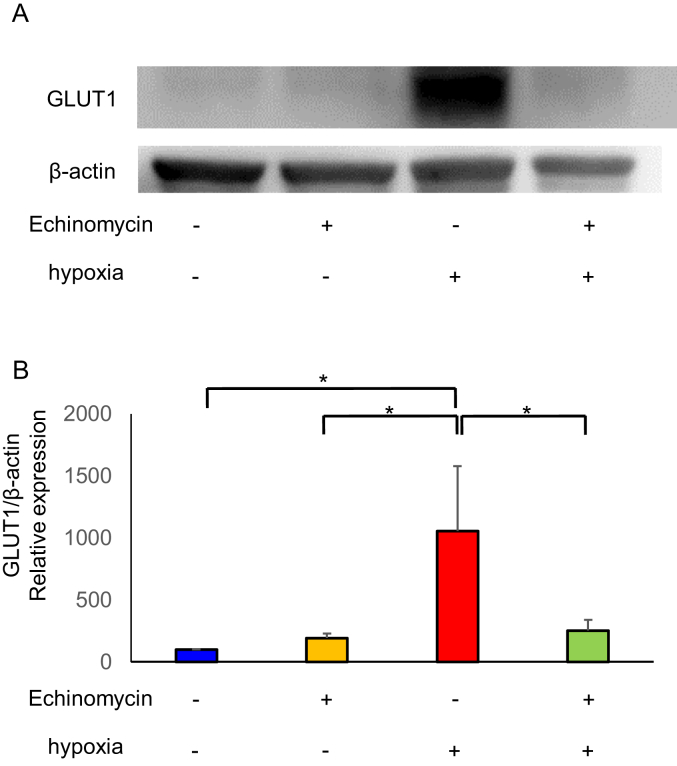
Western blotting analysis for glucose transporter (GLUT) 1 protein expression. The protein obtained from human endometrial stromal cells (hESCs) cultured with or without echinomycin under hypoxic (2% O_2_) or normoxic condition (20% O_2_) for 48 h (A). The protein level of GLUT1 was quantified with ImageJ (B). Results are combined data of three experiments with different cell preparation. Columns and vertical bars represent the mean ± SEM for combined data; ∗P < 0.05. Original non-adjusted Western blotting analysis is shown in Supplementary Material.

To characterize the role of HIF-1α in GLUT1, we used 100 nM concentration of echinomycin [4], a small-molecule inhibitor of HIF-1α activity. It has been reported that echinomycin specifically inhibits DNA binding of HIF-1α by directly interacting with the core sequence within the hypoxia-response element (HRE) of the target gene. GLUT1 protein levels were investigated under normoxia and hypoxia with or without echinomycin in hESCs. The echinomycin significantly reduced GLUT1 expression under the hypoxia (Figure 4; P < 0.05). These indicate that HIF-1α plays a major role in regulating GLUT1 expression under hypoxia in hESCs.

### 3.3 Effects of siRNA-mediated GLUT1 downregulation on mRNA and protein levels

**Figure 5:**
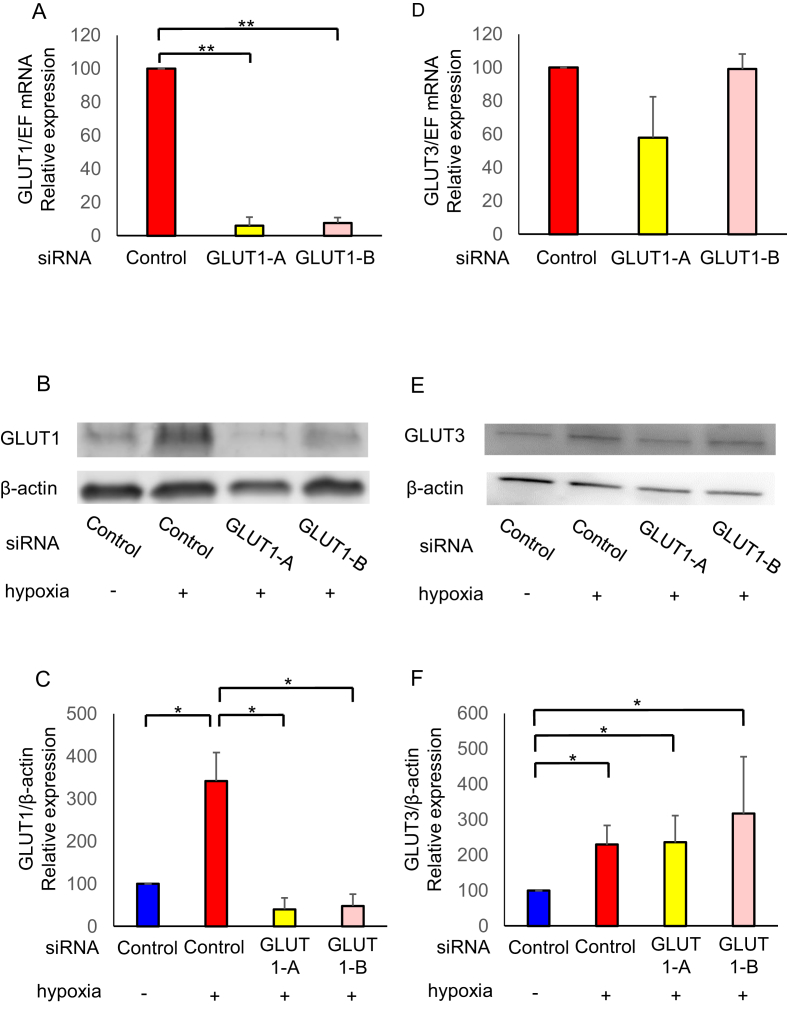
Effects of silencing glucose transporter (GLUT) 1. The human endometrial stromal cells (hESCs) were transfected with GLUT1 small interfering RNA (siRNA; GLUT1-A or GLUT1-B) or non-silencing RNA (Control) and were cultured with 2% O_2_ for 24 h. The mRNA levels of GLUT1 (A), GLUT3 (D) were analyzed by real-time qPCR and normalized to elongation factor1α (EF1α) mRNA expression under hypoxia (2% O_2_). The protein levels of GLUT1 (B), GLUT3 (E) and β-actin were analyzed by western blotting. Total cell lysates were obtained from hESCs cultured under hypoxia (2% O_2_) or normoxia (20% O_2_) for 24 h. The protein levels of GLUT1(C) and GLUT3 (F) was quantified with ImageJ. Results are combined data of three experiments with different cell preparation. Columns and vertical bars represent the mean ± SEM for combined data; ∗P < 0.05. Original non-adjusted Western blotting analysis is shown in Supplementary Material.

**Figure 6:**
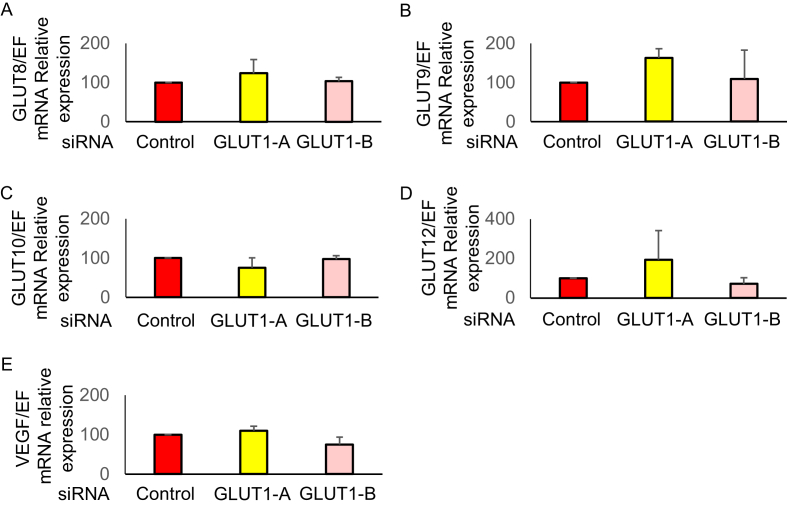
Effects of silencing glucose transporter (GLUT) 1 on the other GLUTs and vascular endothelial growth factor (VEGF). The human endometrial stromal cells (hESCs) were transfected with GLUT1 small interfering RNA (siRNA; GLUT1-A or GLUT1-B) or non-silencing RNA (Control) and were cultured with 2% O_2_ for 24 h. The mRNA levels of GLUT8 (A), GLUT9 (B), GLUT10 (C), GLUT12 (D), and VEGF (E) were analyzed by real-time qPCR and normalized to elongation factor1α (EF1α) mRNA expression under hypoxia (2% O_2_). Results are combined data of three experiments with different cell preparation. Columns and vertical bars represent the mean ± SEM for combined data; ∗P < 0.05.

GLUT1 and GLUT3, mRNA as well as protein levels significantly increased under hypoxia compared to normoxia. Under hypoxia, knockdown by GLUT1-A or GLUT1-B siRNA significantly reduced GLUT1 mRNA (Figure 5A; P < 0.05) and protein levels (Figure 5C; P < 0.05). Notably, silencing GLUT1 did not show significant effect on GLUT3 mRNA (Figure 5D) and protein levels (Figure 5F), as well as other GLUTs, and vascular endothelial growth factor (VEGF) mRNA level under hypoxia (Figure 6). VEGF is also found to be induced by HIF-1α [4].

### 3.4 siRNA-mediated GLUT1 downregulation reduces glucose uptake in hESCs

**Figure 7:**
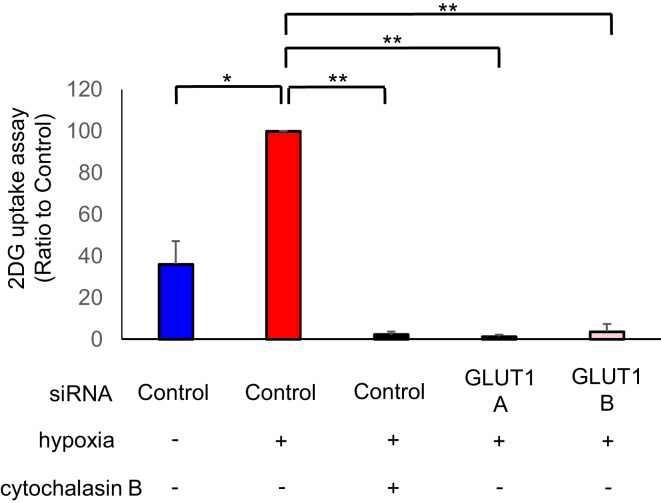
The effect of downregulated glucose transporter (GLUT) 1 on glucose uptake in human endometrial stromal cells (hESCs). Cells were transfected with GLUT1-A or GLUT1-B siRNA or non-silencing control and were cultured under hypoxia (2% O_2_) or normoxia (20% O_2_) for 24 h with or without cytochalasin B. The volume of glucose uptake was measured with 2-deoxyglucose uptake assay. Results are combined data of three experiments with different cell preparations and each value represents mean ± SEM; ∗P < 0.001, ∗∗P < 0.0001.

In order to evaluate the importance of GLUT1 in glucose uptake, a glucose uptake assay was performed using hESCs knocked down GLUT1. As shown in Figure 7, silencing GLUT1 significantly reduced the glucose uptake (Figure 7; P < 0.0001). Cytochalasin B, an inhibitor of GLUTs, significantly reduced the glucose uptake (Figure 7; P < 0.0001). Silencing GLUT1 as well as treatment with cytochalasin B reduced glucose uptake under hypoxia.

### 3.5 Effects of siRNA-mediated GLUT1 downregulation on cell proliferation and apoptosis

**Figure 8:**
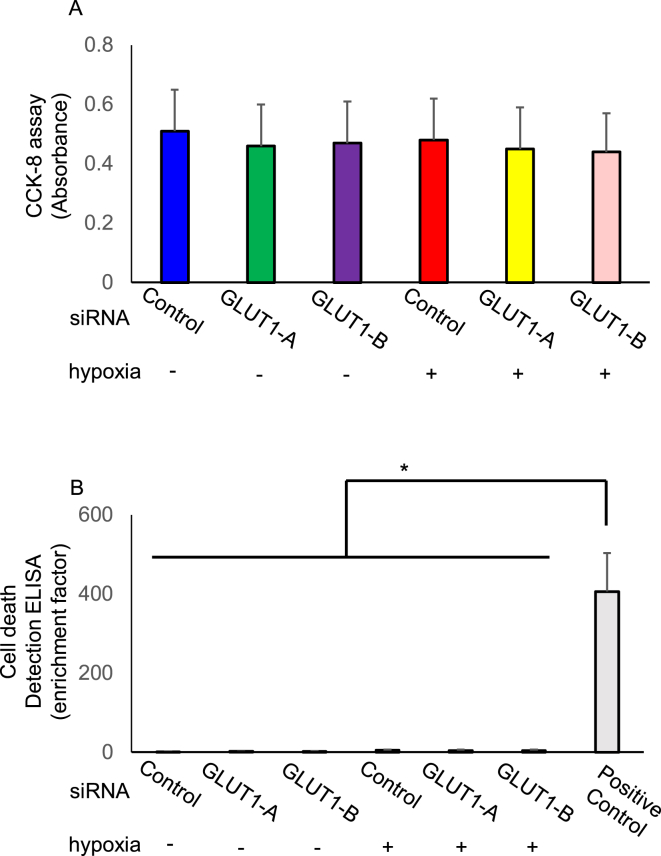
Effects of silencing GLUT1 on cell proliferation and apoptosis. The human endometrial stromal cells (hESCs) were transfected with GLUT1-A or GLUT1-B siRNA or non-silencing control and were cultured under hypoxia (2% O_2_) or normoxia (20% O_2_) for 24 h. The hESCs were added WST-8 (10μL) solution and then read at 450 nm (A). Apoptosis were determined by death detection ELISA (B). Results are combined data of three experiments with different cell preparations and each value represents mean ± SEM; ∗P < 0.001.

To determine the effects of GLUT1 on the regulation of hESC proliferation, the viability of hESCs was analyzed under hypoxia or normoxia for 24 h following the treatment with GLUT1 siRNA (10 nmol/L) for 2 days. A graphic representation of the data showed a linear relationship between cell number and absorbance. The silencing of GLUT1 had no effect on the levels of cell proliferation under hypoxia (Figure 8A).

We further investigated whether a reduction in GLUT1 causes apoptosis. The hESCs were prepared similarly to CCK-8. Enrichment factor is the value of dead cells divided by untreated cells, which correlates with apoptosis. The silencing of GLUT1 had no effect on the levels of apoptosis under hypoxia (Figure 8B).

## 4 Discussion

This study investigated the metabolic alterations in hESCs under the hypoxic environment, and the role of GLUTs underlying these changes. Our results show that hypoxia promotes glycolysis in hESCs. Moreover, GLUT1 is proposed as a critical player in glycolysis because its expression increases glucose uptake in the cell. In summary, hypoxic environment enhances the expression of GLUT1 in hESCs, import large quantities of extracellular glucose, and promote glycolysis for energy production.

To our knowledge, our group is the first to reveal the importance of glycolysis under hypoxic conditions in hESCs. Instead of suppressing the tricarboxylic acid cycle as previously reported [21], hypoxia significantly increased the volume of glucose-6-phosphate, fructose-6-phosphate and fructose-1,6-diphosphate, which are glycolytic intermediates. Most of the glucose is known to be phosphorylated to glucose-6-phosphate as soon as it enters the cell, in order to prevent its diffusion. Due to the charge introduced by phosphorylation, glucose-6-phosphate cannot easily cross the cell membrane. When energy and carbon skeleton are needed, glucose-6-phosphate enters glycolysis. Glucose-6-phosphate is first converted to fructose-6-phosphate by glucose phosphate isomerase. By addition of another phosphate group, which is an irreversible reaction, fructose-6-phosphate to fructose-1,6-diphosphate. Therefore, this reaction irreversibly degrades glucose and produces energy for ATP production through glycolysis. Hence, the hESCs gain energy from glycolysis under hypoxic conditions. Glycolysis is highly conserved biochemical pathway, considered as the most primitive metabolic system, that converts high binding energy contained in glucose into organic acids that can be easily used by organisms, such as pyruvate. Of note, glycolysis occurs also under anaerobic conditions.

HIF-1α regulates this pathway through transcriptional activation of genes encoding glucose transporters and glycolytic enzymes [22]. The GLUT1 promoter region has hypoxia response element where HIF-1α and target gene can bind [23]. A 5′-RCGTG-3′ region of the GLUT1 promoter is HIF-1α binding sequence [24]. We demonstrated that echinomycin, which inhibits HIF-1α binding to hypoxia response element, downregulated GLUT1 under hypoxia (Figure 4). This indicates that GLUT1 acts via HIF-1α. Our study demonstrated that knockdown of GLUT1 in hESCs significantly reduced glucose uptake by 90% or more (Figure 7), suggesting that this transporter plays a major role in glucose uptake. Consistent with these findings, a previous study indicated that GLUT1-shRNA in head and neck squamous cell carcinoma cell line reduced the glucose uptake by 50% [25]. GLUT1 is widely expressed in fetal tissues [10], while it is most frequently expressed in erythrocytes and in endothelial cells of the blood-brain barrier in adults [26]. However, it is thought to be involved in minimal glucose uptake in all cells. As previously reported, GLUT1-deficiency syndrome leads to a metabolic encephalopathy caused by the inability of the central nervous system to take up glucose [27]. Consequently, ketone is used instead of glucose as the alternate source of energy in the brain [28]. In hESCs, we demonstrated that GLUT1 deficiency prevents glucose uptake without affecting apoptosis and cell toxicity. The inhibition of GLUT1 in cancer stem cells reduces self-renewal and tumor initiation capacity, but is not biologically harmful to host animals [29]. Previous reports have shown that HIF-1α and its downstream gene GLUT1 are increasingly expressed from normal to endometrial hyperplasia, further to endometrial adenocarcinoma [30]. Thus, elucidating the common features of GLUT1 overexpression in hESCs under hypoxia and in tumor cells may lead to therapeutic strategies in the future.

*von Wolff M et al.* reported that GLUT1 expression was enhanced in hESCs during early pregnancy [31] and decidualized hESCs were shown to have increased glucose uptake [32]. Importantly, during decidualization, a simultaneous upregulation of GLUT1 mRNA [12] and protein expression [33] was also reported. In complementary results, the downregulated expression of GLUT1 on ESCs led to a reduced glucose uptake and suppressed decidualization [34]. Collectively, these results suggest that GLUT1 is necessary for implantation and maintenance of pregnancy. However, further investigations are required for complete elucidation of the role played by GLUT1 in decidualization.

The present study showed that GLUT3 was also induced in hypoxic environment. In endothelial cells of human umbilical vein, knockdown of GLUT3 decreased glucose uptake by 40% under hypoxia [35]. GLUT3 has high affinity for glucose, and is present in tissues with a high demand for glucose as a fuel, especially in the brain [36]. Moreover, GLUT3 was detected in first trimester human placenta [37]. There was no report of endometrial GLUT3 expression during menstruation. Combined with our findings, it is necessary to clarify the role of GLUT3 under hypoxia in the future. Clinically, GLUT3-deficiency syndrome has not been reported yet. In endometriosis, where ESCs were ectopically engrafted and proliferated, GLUT3 was significantly increased compared to eutopic ESCs [38]. This was consistent with the significant increase of GLUT3 in hESCs under hypoxia. Future studies on GLUT3 will help to elucidate its role in the development of endometriosis.

On the contrary, in this study, only GLUT8 mRNA was found to be reduced under hypoxia. Previous reports have shown that GLUT8 is also significantly downregulated in bovine mammary epithelial cells under hypoxia [39]. Whereas the role of GLUT8 is not yet clear. In mouse uterus, GLUT8 appears to be maximum during non-pregnant uterus estrus, with a dramatic increase during embryo implantation and stromal decidualization [40]. Other GLUTs are constitutively located on the cell surface or move between cell storage compartments and the cell surface in response to stimuli such as insulin and high glucose. Interestingly, GLUT8 is localized into the endoplasmic reticulum, where it may provide the glucose necessary for glycosylation of proteins during decidualization [41]. Our results suggest that suppressing GLUT8 under hypoxic conditions may promote the preferential use of glucose for glycolysis. In one study, GLUT8 knockout mice showed a very mild phenotype. Hippocampal neuron proliferation and a slight increase in P-wave duration on the electrocardiogram were observed [42]. This suggests that GLUT8 deficiency is not a major biological problem in human.

Finally, the behavior of hESCs under hypoxic conditions is also of great importance during the implantation period. In previous studies carried out in mice, implantation failure occurred when knocking down HIF-2α in ESC [43]. This indicates the importance of the induction of HIF in ESCs during the implantation stage.

## 5 Conclusion

We demonstrated that hypoxia is involved in the regulation of metabolic pathways and GLUT expression in hESCs. In hypoxic environment, hESCs increased extracellular glucose uptake, enhanced glycolysis, and thereby obtained energy. This was achieved by enhancing the expression of GLUT1. These findings may be relevant for menstrual and implantation period. But low oxygen concentration during menstrual and implantation period is unclear, which is the limitation of this study. Next, we need to experiment with the organization. Better understanding of glucose metabolism in the endometrium would give crucial information for regenerative medicine, and etiology of severe diseases such as endometriosis and endometrial tumor.

## Declarations

### Author contribution statement

T. Kido: Conceived and designed the experiments; Performed the experiments; Analyzed and interpreted the data; Contributed reagents, materials, analysis tools or data; Wrote the paper.

H. Okada: Conceived and designed the experiments; Analyzed and interpreted the data.

H. Murata: Performed the experiments; Analyzed and interpreted the data; Contributed reagents, materials, analysis tools or data.

A. Nishigaki: Performed the experiments; Contributed reagents, materials, analysis tools or data.

H. Tsubokura, S. Komiya, N. Kida, M. Kakita-Kobayashi, Y. Hisamatsu, T. Tsuzuki, Y. Hashimoto: Performed the experiments.

### Funding statement

This work was supported by a research grant D2 from 10.13039/501100012412Kansai Medical University, and the 10.13039/501100001691Japan Society for the Promotion of Science KAKENHI (Grant Numbers #JP17K11260 and #JP19K09766).

### Competing interest statement

The authors declare no conflict of interest.

### Additional information

No additional information is available for this paper.

## Appendix A Supplementary data

renamed_abc32.pptx renamed_abc32.pptx
