# Supplementary material for: Glucose transporter 1 is important for the glycolytic metabolism of human endometrial stromal cells in hypoxic environment
Source: Heliyon. 2020 Jun 8;6(6):e03985. doi: 10.1016/j.heliyon.2020.e03985 (PMC7286975; doi:10.1016/j.heliyon.2020.e03985)
Supplement: renamed_abc32.pptx [file mmc1.pptx]

## Slide 1
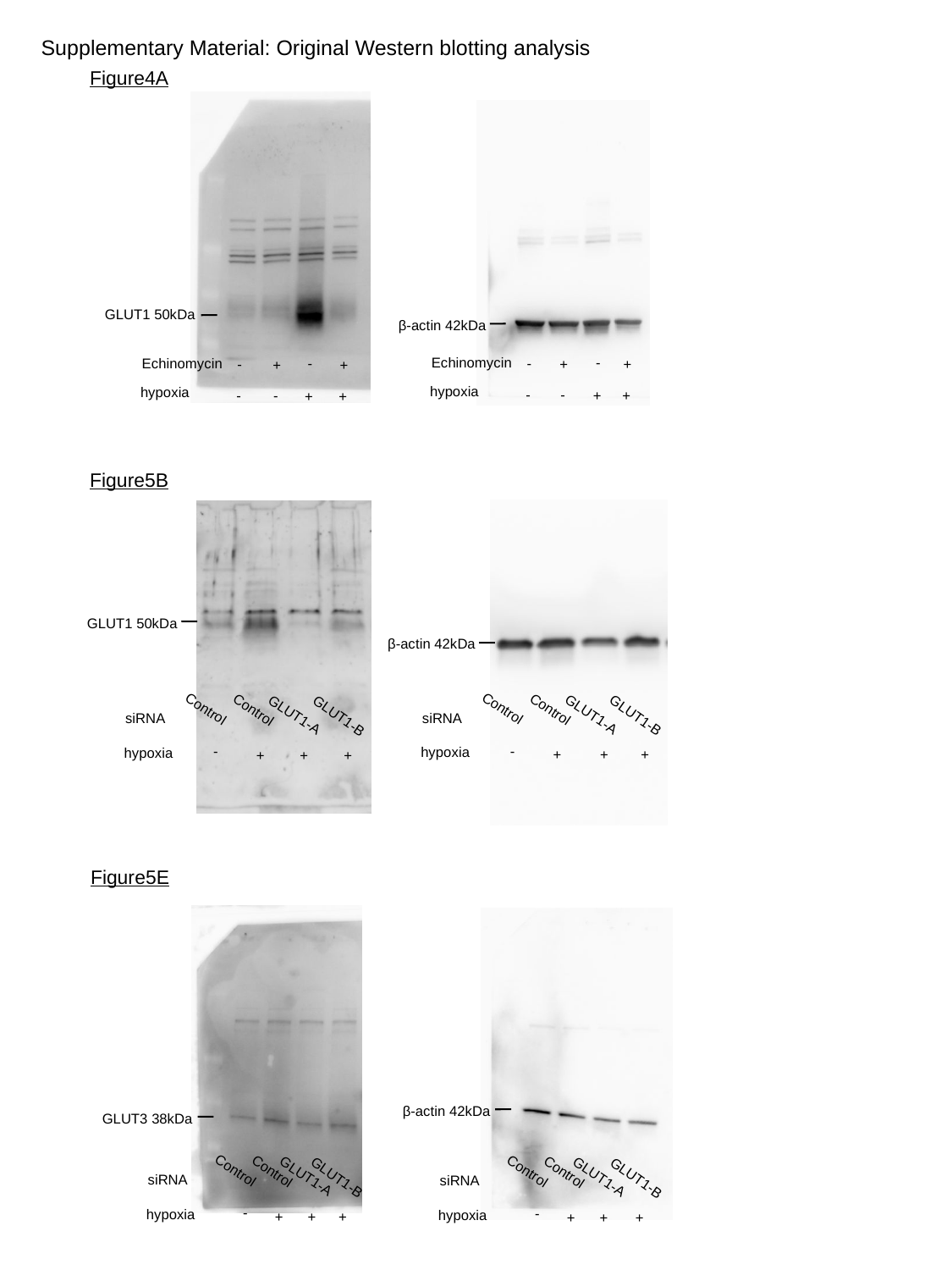

Supplementary Material: Original Western blotting analysis
Figure4A
GLUT1 50kDa
β-actin 42kDa
Echinomycin
-
-
Echinomycin
-
-
+
+
+
+
hypoxia
hypoxia
-
-
-
-
+
+
+
+
Figure5B
GLUT1 50kDa
β-actin 42kDa
Control
Control
Control
Control
siRNA
siRNA
GLUT1-A
GLUT1-A
GLUT1-B
GLUT1-B
-
-
hypoxia
hypoxia
+
+
+
+
+
+
Figure5E
β-actin 42kDa
GLUT3 38kDa
Control
Control
Control
Control
siRNA
siRNA
GLUT1-A
GLUT1-A
GLUT1-B
GLUT1-B
-
-
hypoxia
hypoxia
+
+
+
+
+
+
